# Machine Learning Classification of Cognitive Status in Community-Dwelling Sarcopenic Women: A SHAP-Based Analysis of Physical Activity and Anthropometric Factors

**DOI:** 10.3390/medicina61101834

**Published:** 2025-10-14

**Authors:** Yasin Gormez, Fatma Hilal Yagin, Yalin Aygun, Sarah A. Alzakari, Amel Ali Alhussan, Mohammadreza Aghaei

**Affiliations:** 1 Department of Management Information Systems, Faculty of Economics and Administrative Sciences, Sivas Cumhuriyet University, Sivas 58140, Türkiye; 2 Department of Biostatistics, Faculty of Medicine, Malatya Turgut Ozal University, Malatya 44210, Türkiye; 3 Department of Computer Science, Lakehead University, Thunder Bay, ON P7B 5E1, Canada; 4 Department of Sport Management, Faculty of Sport Sciences, Inonu University, Malatya 44280, Türkiye; 5 Department of Computer Sciences, College of Computer and Information Sciences, Princess Nourah Bint Abdulrahman University, P.O. Box 84428, Riyadh 11671, Saudi Arabia; 6 Department of Ocean Operations and Civil Engineering, Norwegian University of Science and Technology (NTNU), 6025 Alesund, Norway

**Keywords:** machine learning, cognitive impairment, physical activity, SHAP, women, sarcopenia, sedentary behavior, MMSE

## Abstract

*Background and Objectives*: Sarcopenia, characterized by progressive loss of skeletal muscle mass and function, has increasingly been recognized not only as a physical health concern but also as a potential risk factor for cognitive decline. This study investigates the application of machine learning algorithms to classify cognitive status based on Mini-Mental State Examination (MMSE) scores in community-dwelling sarcopenic women. *Materials and Methods*: A dataset of 67 participants was analyzed, with MMSE scores categorized into severe (≤17) and mild (>17) cognitive impairment. Eight classification models—MLP, CatBoost, LightGBM, XGBoost, Random Forest (RF), Gradient Boosting (GB), Logistic Regression (LR), and AdaBoost—were evaluated using a repeated holdout strategy over 100 iterations. Hyperparameter optimization was performed via Bayesian optimization, and model performance was assessed using metrics including weighted F1-score (w_f1), accuracy, precision, recall, PR-AUC, and ROC-AUC. *Results*: Among the models, CatBoost achieved the highest w_f1 (87.05 ± 2.85%) and ROC-AUC (90 ± 5.65%), while AdaBoost and GB showed superior PR-AUC scores (92.49% and 91.88%, respectively), indicating strong performance in handling class imbalance and threshold sensitivity. SHAP (SHapley Additive exPlanations) analysis revealed that moderate physical activity (moderatePA minutes), walking days, and sitting time were among the most influential features, with higher physical activity associated with reduced risk of cognitive impairment. Anthropometric factors such as age, BMI, and weight also contributed significantly. *Conclusions*: The results highlight the effectiveness of boosting-based models in capturing complex patterns in clinical data and provide interpretable evidence supporting the role of modifiable lifestyle factors in cognitive health. These findings suggest that machine learning, combined with explainable AI, can enhance risk assessment and inform targeted interventions for cognitive decline in older women.

## 1. Introduction

Cognitive impairment represents a critical public health challenge as the global population ages, with profound implications for healthcare systems and individual quality of life. Age-related cognitive decline, affecting millions of older adults worldwide, is driven by biological processes including mitochondrial dysfunction, cellular senescence, and chronic inflammation, as well as neuropathological lesions characteristic of dementia [1,2]. The Mini-Mental State Examination (MMSE) remains the most widely used screening tool for cognitive assessment, providing standardized evaluation across orientation, memory, attention, language, and visuospatial domains, particularly valuable for community-dwelling older populations [1,2].

Emerging evidence reveals complex interrelationships between physical health, body composition, and cognitive function. Sarcopenia—progressive loss of skeletal muscle mass and function—has been increasingly recognized as a potential risk factor for cognitive decline, with cognitive impairment prevalence 30–50% higher among sarcopenic versus non-sarcopenic older adults [3,4,5]. The ‘sarcopenia-cognitive impairment’ hypothesis proposes shared biological mechanisms including chronic inflammation, oxidative stress, mitochondrial dysfunction, and dysregulation of neurotrophic factors. Physical activity has emerged as a promising modifiable factor, with moderate-intensity exercise demonstrating protective effects against both conditions through enhanced cerebral blood flow, neurogenesis, reduced inflammation, and improved insulin sensitivity [6,7].

Despite growing recognition of these relationships, critical knowledge gaps remain. First, the specific patterns and intensities of physical activity that most strongly influence cognitive status in older women—a population particularly vulnerable to both sarcopenia and cognitive decline—have not been adequately characterized. Second, the complex, potentially nonlinear interactions between physical activity patterns, sedentary behavior, and anthropometric variables require analytical approaches beyond traditional statistical methods. Third, while machine learning techniques offer powerful tools for identifying patterns in multidimensional datasets, their “black box” nature has limited clinical interpretability and implementation [8,9].

Recent advances in explainable artificial intelligence (XAI), particularly SHapley Additive exPlanations (SHAP), provide model-agnostic methods for interpreting machine learning predictions by quantifying each feature’s contribution to model output [10]. This approach enables transparent identification of modifiable factors most strongly associated with cognitive outcomes, addressing the interpretability challenge while maintaining analytical sophistication.

The present study addresses these gaps by applying multiple machine learning algorithms with SHAP-based interpretation to classify cognitive status in community-dwelling older women. Specifically, we examine: (1) which aspects of physical activity and sedentary behavior most strongly predict cognitive function; (2) how these factors interact with anthropometric variables; and (3) whether the protective association between moderate physical activity and sarcopenia extends to cognitive health outcomes. Our approach advances beyond previous research by capturing the complex interplay between physical activity, body composition, and cognitive function through a comprehensive, interpretable machine learning framework. We employ Bayesian hyperparameter optimization to enhance model performance and utilize SHAP values for clinically actionable risk assessment. These findings have important implications for developing targeted, evidence-based interventions to preserve cognitive health in older women, supporting physical activity assessment and promotion as integral components of cognitive health strategies for aging populations.

## 2. Materials and Methods

### 2.1. Population, Dataset, and Ethical Procedures

The current study, using publicly available data from a cross-sectional study using a quantitative descriptive approach, included data from community-dwelling older women aged ≥60 years with sarcopenia [11]. The Inonu University Health Sciences Non-Interventional Clinical Research Ethics Committee approved this study (approval number: 2025/7305, 11 March 2025). Inclusion criteria were female, the ability to walk independently, the absence of unstable cardiovascular conditions, acute infections, back pain, or tumors, and no unintentional weight loss of more than 3 kg in the last three months. Participants completed a cognitive assessment questionnaire on the first day, and PA and sarcopenia results were measured the following day. The MMSE test was administered to identify cognitive impairment in older women. Data from women with a score of 12 or less were excluded from the analysis. The short version of the International Physical Activity Questionnaire (IPAQ) was used to assess participants’ physical activity levels [11].

This study analyzed physical activity and anthropometric factors to classify cognitive status in community-dwelling older women based on MMSE scores. Below is a concise summary of the key risk factors and types of cognitive impairment considered:

- Physical Activity Factors
  ○ Moderate physical activity duration (moderatePA minutes): Weekly duration of moderate-intensity physical activity (in minutes).
  ○ Walking days (walk days): Number of days per week spent walking.
  ○ Sitting time (sitting time minutes, 7 days): Total weekly sitting time (in minutes), as an indicator of sedentary behavior.
- Anthropometric Factors
  ○ Age: Age of participants (in years).
  ○ Body Mass Index (BMI): Calculated as weight divided by the square of height (kg/m^2^).
  ○ Weight: Body weight of participants (in kg).
  ○ Height: Height of participants (in cm).
- Types of Cognitive Impairment
  ○ Severe cognitive impairment: Individuals with MMSE scores ≤ 17.
  ○ Mild cognitive impairment: Individuals with MMSE scores > 17.

### 2.2. Classification Models

Classification is a supervised machine learning task that aims to assign observations to one of several predefined categorical classes. In this study, various algorithms were employed to solve the classification problem. The multilayer perceptron (MLP) neural network stands out for its ability to learn complex nonlinear relationships [11]. Gradient boosting-based algorithms such as CatBoost [12], LightGBM [13], and XGBoost [8] provide high performance, especially when working with categorical variables and large datasets. Random Forest (RF) [14], and Gradient Boosting (GB) [15] algorithms rely on ensembles of decision trees to balance bias and variance. Logistic regression (LR) [16] is a widely used baseline method for modeling binary outcomes. AdaBoost [17] aims to improve prediction accuracy by sequentially boosting weak classifiers. In this study, these models were comparatively evaluated, and classification performance was assessed using various metrics. All computational analyses in this research were conducted using Python 3.10 within the Spyder 6.0.7 platform. This integrated development environment provides an interactive framework for data analysis and model construction.

### 2.3. Performance Metrics

Performance metrics are quantitative measures used to evaluate how well a machine learning model performs on a given task. They are crucial for assessing not only the overall predictive capability of the model but also for understanding its strengths and limitations across different aspects of classification performance. In this study, we utilized weighted F1-score (w_f1), accuracy (acc), precision (pre), recall (rec), precision-recall curve score (pr_auc), and roc_auc score to comprehensively assess the classification models, capturing both threshold-dependent and threshold-independent performance characteristics. Detailed calculations and explanations of the metrics used can be found in the relevant references [18,19,20,21].

### 2.4. Bayesian Optimization

Bayesian optimization is a sequential model-based optimization technique that efficiently finds the global optimum of expensive, black-box functions by building a probabilistic surrogate (typically a Gaussian process) to model the objective and selecting new points via an acquisition function that balances exploration and exploitation. Unlike grid or random search, it strategically uses prior evaluations to predict promising regions, often requiring far fewer evaluations to locate optima. This makes it particularly effective for hyperparameter tuning in machine learning. For a detailed explanation of the method, please refer to the cited article [22].

### 2.5. SHapley Additive exPlanations

SHAP is a unified framework that explains machine learning model predictions by assigning each feature a Shapley value, derived from cooperative game theory. These values quantify how much each feature contributes to pushing a prediction away from the dataset’s average output. SHAP ensures properties like local accuracy (the contributions sum to the model output), consistency, and missingness. It offers both model-agnostic (e.g., Kernel SHAP) and model-specific (e.g., TreeSHAP) algorithms for efficient computation. For a detailed explanation of the method, please refer to the cited article [23,24].

## 3. Results

**Figure 1:**
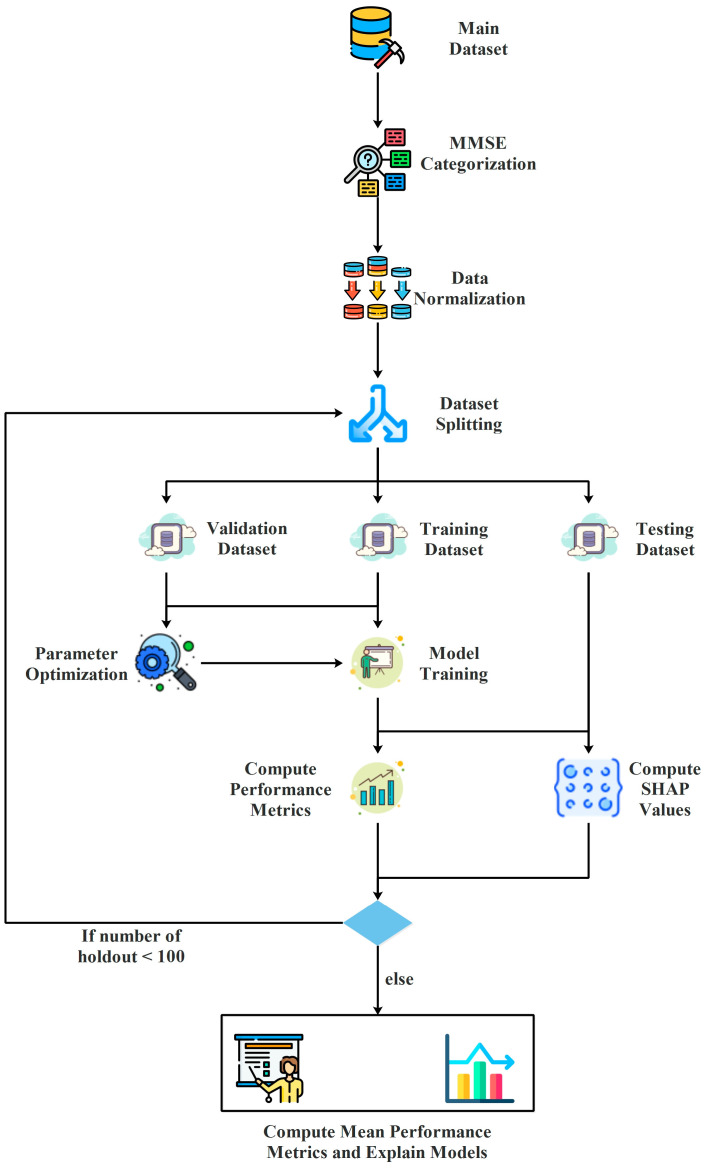
Step-by-Step Process of the Machine Learning Experiments Conducted for Data Analysis and Model Evaluation.

As illustrated in Figure 1, the machine learning experimental workflow of this study comprises several sequential steps. First, the class label in the dataset (MMSE) was categorized, followed by data normalization. The dataset was then split into three subsets: training, testing, and validation. Hyperparameter optimization was performed using the training and validation sets through a Bayesian optimization approach, and the resulting optimal hyperparameters were employed to train the model on the training set. Subsequently, performance metric scores and SHAP values were calculated on the test set using the trained model. Since a repeated holdout strategy was adopted, this entire process was executed 100 times, after which the average metric scores and model explanations were reported.

### 3.1. Data Preparation

In the initial phase of dataset preparation, the MMSE score, originally provided as a continuous variable, was categorized. In this process, guided by findings from previous studies in the literature, samples with an MMSE score of 17 or below were labeled as severe cognitive impairment, while those with a score above 17 were labeled as mild cognitive impairment. The MMSE cut-off of 17 was selected based on Whelan et al. (2009), who identified this threshold as optimal for distinguishing severe cognitive impairment [25]. Following the categorization, data normalization was performed to ensure that all features contributed equally to the model, thereby promoting fairer and more accurate training. For this purpose, the MinMaxScaler function from the sklearn library in Python version 3.10 was employed. At the end of this stage, the resulting dataset comprised 67 samples, with 28 (42.8%) belonging to severe cognitive impairment and 39 (57.2%) to mild cognitive impairment. Although the class sizes were not perfectly balanced, the relatively small difference led to the expectation that this level of imbalance would not pose significant issues during model training. Indeed, prior research suggests that class distributions within the 60–40% range are generally considered to represent mild imbalance, which in most cases does not substantially affect model performance [26].

### 3.2. Hyperparameter Optimization

After preparing the dataset for training, the hyperparameter optimization phase was initiated. In this step, 10% of the samples from the entire dataset were randomly selected to form the validation set, while 20% were randomly selected to constitute the test set. The remaining samples were allocated to the training set. To ensure that the class distribution was preserved across the training, testing, and validation sets, the train_test_split function from the sklearn library was utilized with the stratify parameter. As a result, the training set contained 46 samples, the test set 14 samples, and the validation set 7 samples. During the model optimization phase, several specific library versions were employed to ensure reproducibility and stability of the results. Bayesian optimization was performed using the scikit-optimize (skopt) library version 0.9.0. For developing the XGBoost model, xgboost version 2.0.3 was used, while the LightGBM and CatBoost models were implemented with lightgbm version 4.6.0 and catboost version 1.2.5, respectively. Additionally, other classification models were developed using scikit-learn (sklearn) version 1.5.0. The optimized hyperparameter values for each model are presented in Table 1.

As presented in Table 1, different hyperparameters of the proposed models were optimized. Since the hyperparameter optimization process was repeated in each holdout iteration, each model has 100 distinct sets of optimal hyperparameters. Therefore, these optimal hyperparameters are not included in Table 1. For each model, the remaining hyperparameters not shown in Table 1 were kept at their default values as specified in the respective libraries used during model development. During the hyperparameter optimization phase, the w_f1 computed on the validation dataset was used as the performance metric. In the skopt library, the acquisition function parameter was set to Expected Improvement, and the Bayesian optimization process was executed for 25 calls.

### 3.3. Model Training and Evaluation

Upon completion of hyperparameter optimization, each model was trained with the identified optimal hyperparameters during every repeated holdout iteration. Subsequently, model performances were evaluated on the testing dataset employing a range of metrics. Given that performance metrics were computed separately for each holdout, this process yielded 100 distinct scores per metric for each model. Table 2 summarizes these results by presenting the mean and the corresponding standard deviation (std) for each metric.

The table presents the comparative performance of eight different machine learning models (MLP, CatBoost, LightGBM, XGBoost, RF, GB, LR, and AdaBoost) in classifying MMSE status, evaluated over 100 repeated holdout experiments. Each cell reports the mean ± std for various metrics: w_f1, acc, pre, rec, pr_auc, and auc_roc, all expressed as percentages. According to the results in this table, CatBoost, GB, and AdaBoost consistently outperformed other models across most metrics. CatBoost achieved the highest mean w_f1 (87.05 ± 2.85%), closely followed by AdaBoost (86.43 ± 2.15%) and GB (86.35 ± 2.14%). Similarly, these three models also reported the highest accuracies. All models maintained a reasonable balance between precision and recall. Notably, GB and AdaBoost achieved high precision (88.58% and 88.32%, respectively) while maintaining high recall (both 86.42%), indicating stable sensitivity and specificity. AdaBoost and GB reported the highest pr_auc (92.49 ± 5.83% and 91.88 ± 5.18%, respectively), suggesting superior ability to differentiate between classes under varying decision thresholds. CatBoost followed with 89.6 ± 8.48%. Regarding roc_auc, CatBoost led with 90 ± 5.65%, slightly ahead of AdaBoost and GB. The std indicate the models’ robustness under repeated sampling. CatBoost, GB, and AdaBoost exhibited low variability across most metrics (std < 3% for w_f1 and acc), emphasizing their stable generalization. In contrast, RF showed relatively higher variability (e.g., w_f1 std of 6.68%), implying greater sensitivity to training data splits. The MLP, LightGBM, XGBoost, and LR demonstrated lower overall performance, with w_f1 ranging from ~77% to ~80%, though still maintaining acceptable classification levels. Interestingly, XGBoost’s pr_auc (85.93 ± 10.79%) was somewhat higher than its roc_auc (81.88 ± 9.4%), suggesting potential calibration issues or threshold sensitivity. In summary, ensemble-based tree models, particularly CatBoost, GB, and AdaBoost, provided superior and more consistent performance for MMSE status classification in this study. These findings highlight the effectiveness of boosting approaches in handling structured clinical data with possible nonlinear patterns.

### 3.4. Model Explanations

**Figure 2:**
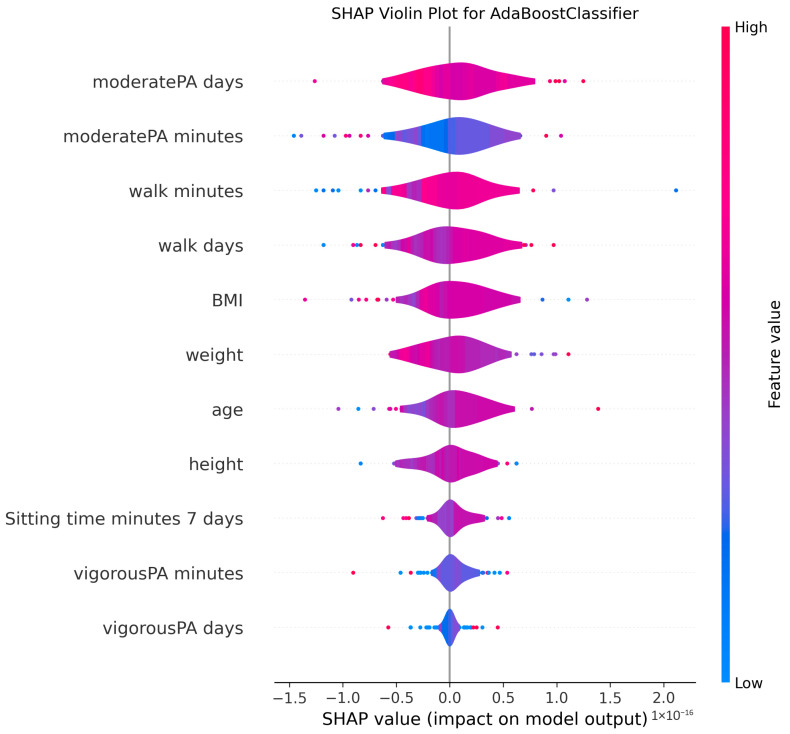
SHAP summary violin plots indicating feature importance across different machine learning models for MMSE classification for AdaBoost Classifier. Plots are generated using SHAP values on test datasets from 100 repeated holdout experiments.

**Figure 3:**
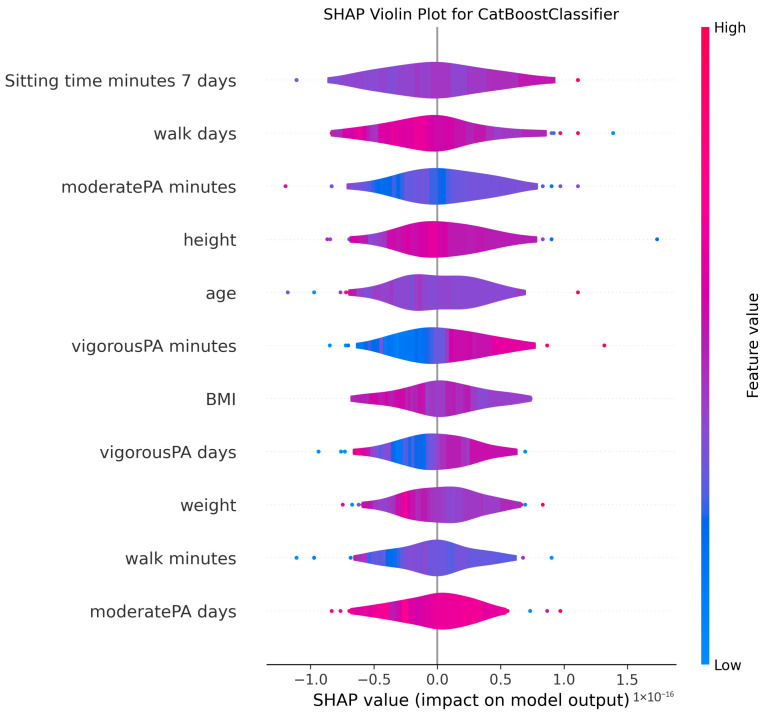
SHAP summary violin plots indicating feature importance across different machine learning models for MMSE classification for CatBoost Classifier. Plots are generated using SHAP values on test datasets from 100 repeated holdout experiments.

**Figure 4:**
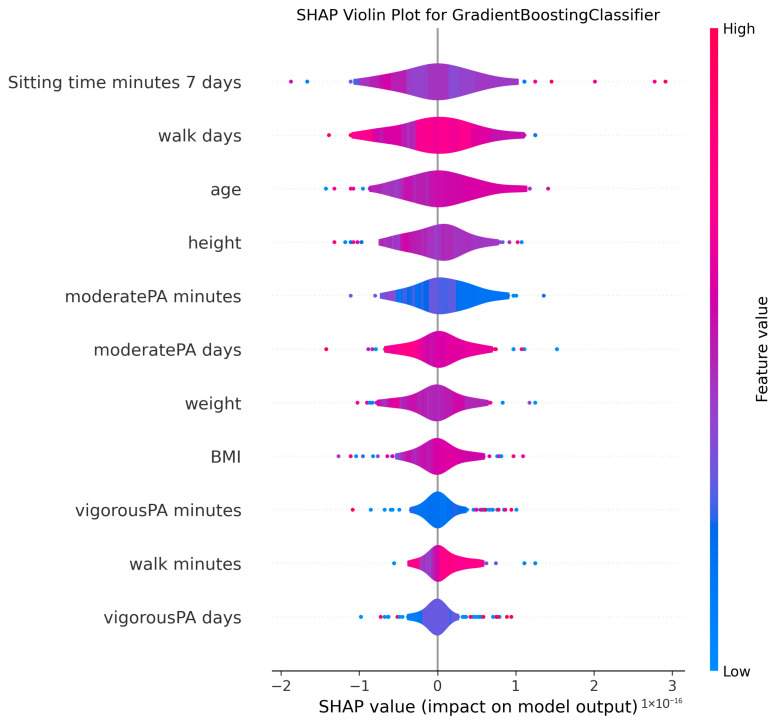
SHAP summary violin plots indicating feature importance across different machine learning models for MMSE classification for Gradient Boosting Classifier. Plots are generated using SHAP values on test datasets from 100 repeated holdout experiments.

**Figure 5:**
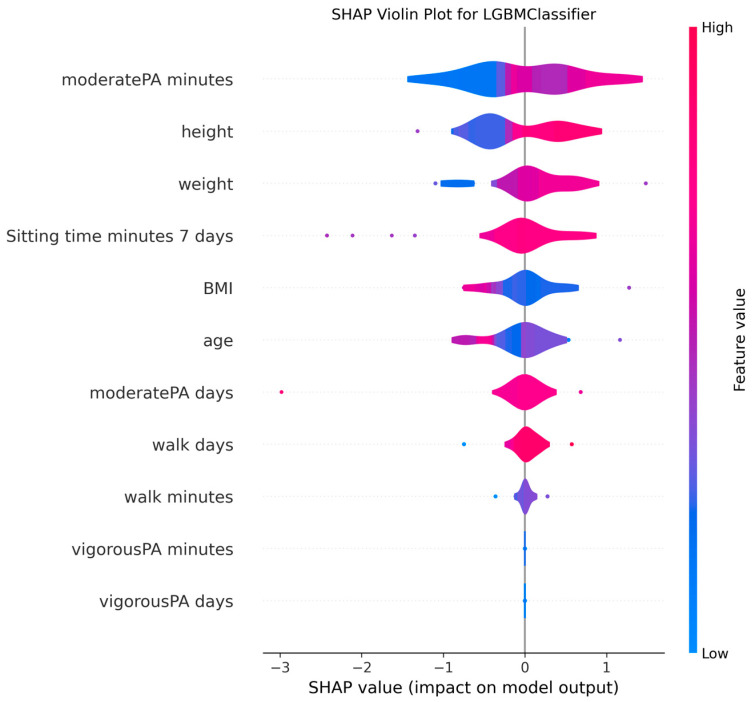
SHAP summary violin plots indicating feature importance across different machine learning models for MMSE classification for LightGBM Classifier. Plots are generated using SHAP values on test datasets from 100 repeated holdout experiments.

**Figure 6:**
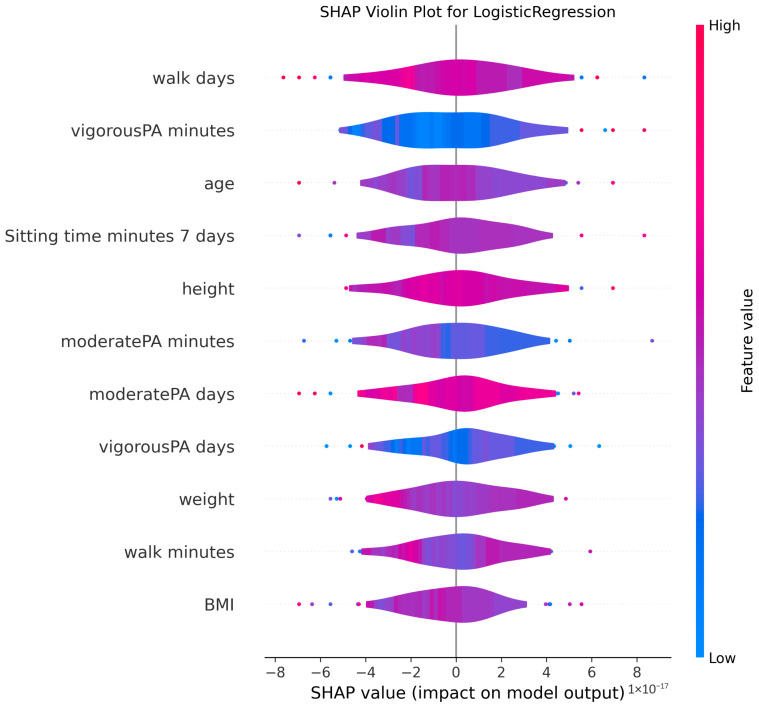
SHAP summary violin plots indicating feature importance across different machine learning models for MMSE classification for Logistic Regression Classifier. Plots are generated using SHAP values on test datasets from 100 repeated holdout experiments.

**Figure 7:**
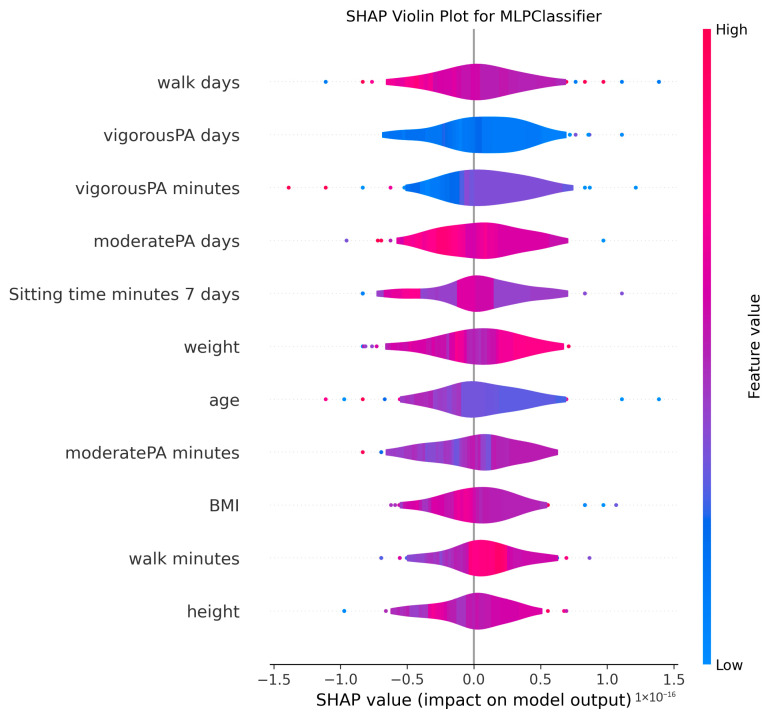
SHAP summary violin plots indicating feature importance across different machine learning models for MMSE classification for MLP Classifier. Plots are generated using SHAP values on test datasets from 100 repeated holdout experiments.

**Figure 8:**
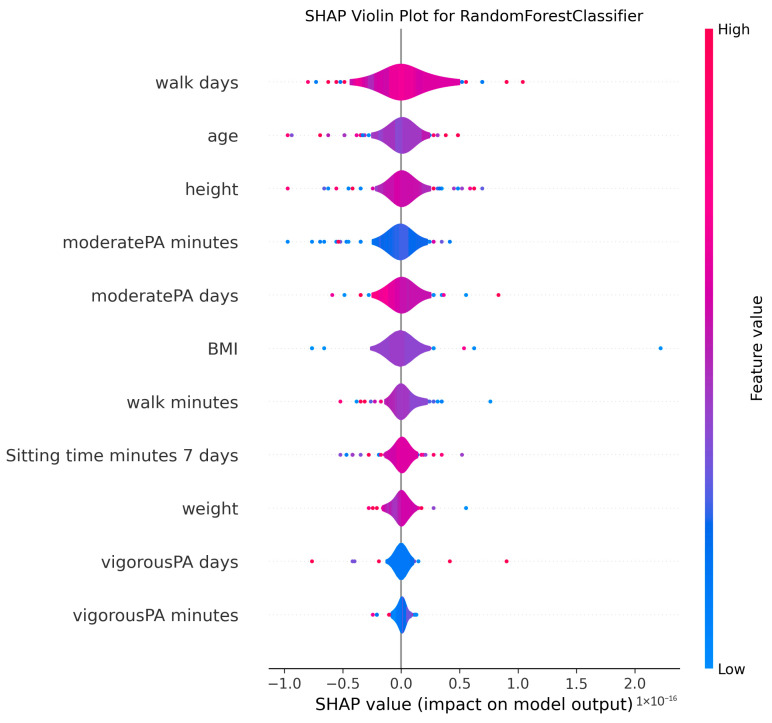
SHAP summary violin plots indicating feature importance across different machine learning models for MMSE classification for Random Forest Classifier. Plots are generated using SHAP values on test datasets from 100 repeated holdout experiments.

**Figure 9:**
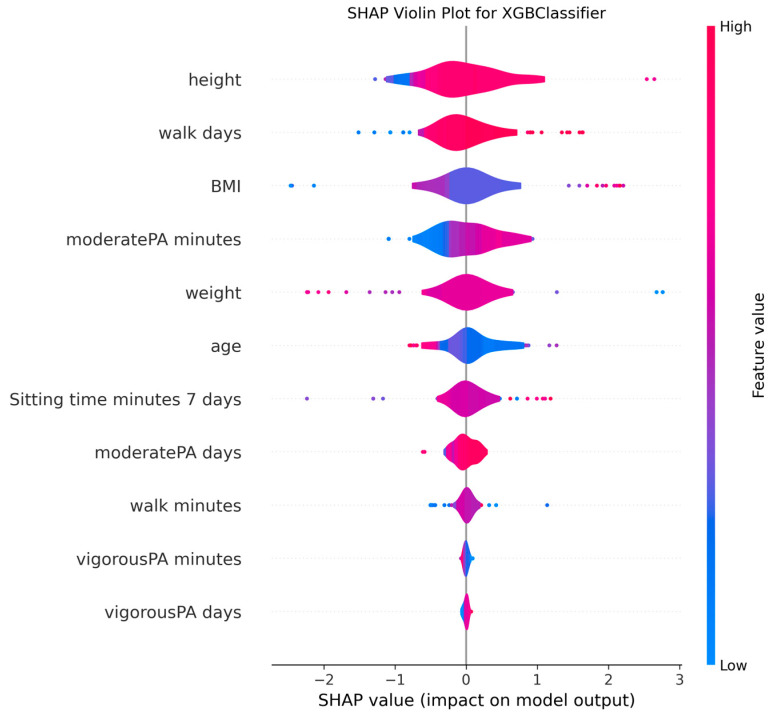
SHAP summary violin plots indicating feature importance across different machine learning models for MMSE classification for XGBoost Classifier. Plots are generated using SHAP values on test datasets from 100 repeated holdout experiments.

The ability to interpret the predictions of machine learning models, particularly those of complex algorithms often referred to as “black-box” models, is becoming increasingly important in both academic research and practical applications. Understanding why a model makes a particular prediction is critical for enhancing the model’s trustworthiness, identifying and mitigating potential biases, diagnosing the root causes of incorrect predictions to improve performance, and deriving business or scientific insights from the findings. Therefore, in this study, the algorithms proposed for MMSE prediction were explained using the SHAP method after the training phase. To facilitate model interpretation, version 0.46.0 of the official SHAP library in Python was utilized. For each method, SHAP values were computed by applying the trained model from each holdout iteration to the corresponding test dataset. These SHAP values from all holdouts were then concatenated to obtain the aggregated SHAP values across all iterations. Finally, violin plots illustrating these aggregated SHAP values are presented in Figure 2, Figure 3, Figure 4, Figure 5, Figure 6, Figure 7, Figure 8 and Figure 9.

The figures display SHAP summary violin plots for each of the eight machine learning models employed to classify MMSE status, providing insight into the contribution and impact of individual features on model predictions. The SHAP values were computed on testing sets from 100 repeated holdout experiments, ensuring robust estimation of feature importance and the consistency of their effects. According to this figure, several features consistently emerged as influential across models, including moderatePA minutes, walk days, Sitting time minutes (7 days), age, height, BMI, and weight. This aligns with known clinical and epidemiological associations between physical activity, anthropometrics, and cognitive function. Notably, moderatePA minutes and walk days were recurrently among the top-ranked features, especially prominent in AdaBoost, LightGBM, and XGBoost, underscoring the relevance of moderate physical activity frequency and walking behavior in differentiating MMSE categories.

Models such as CatBoost, GB, and AdaBoost, which demonstrated superior predictive metrics (e.g., CatBoost with a w_f1 of 87.05% and AdaBoost with the highest pr_auc of 92.49%), also exhibited clear, well-separated SHAP distributions for key features. The CatBoost plot, sitting time, walk days, and moderatePA minutes show distinct SHAP value spreads, reflecting stable, meaningful contributions to model output. Similarly, GB and AdaBoost plots reveal broader SHAP value distributions for top features, indicating that variations in these variables have substantial and consistent impacts on the predicted probability of MMSE impairment.

The color gradients in the violin plots (from blue indicating low feature values to pink indicating high feature values) illustrate how increases in features such as moderatePA minutes and walk days generally lead to positive SHAP values in several models (e.g., XGBoost, AdaBoost), suggesting a protective role (lower risk of cognitive impairment). Conversely, longer sitting time minutes tends to shift SHAP values negatively in models like GB and CatBoost, highlighting its potential adverse influence on MMSE classification.

The LR plot shows more symmetric and uniform SHAP distributions across features, consistent with its simpler linear nature and comparatively lower predictive performance (w_f1 = 77.45%). In contrast, tree-based ensemble models captured more complex, heterogeneous relationships, evident from the wider spread of SHAP values. The RF model exhibited less pronounced SHAP value separation, aligning with its higher standard deviation in performance metrics, suggesting greater sensitivity to data variability. The SHAP analysis validates the interpretability and reliability of the ensemble boosting models (CatBoost, GB, AdaBoost) not only through quantitative performance metrics but also by demonstrating clear, biologically plausible patterns of feature importance. These plots collectively reinforce the central role of moderate-intensity physical activity, walking frequency, and sedentary behavior in influencing cognitive outcomes, providing valuable interpretive support for the clinical implications of the machine learning findings.

## 4. Discussion

### 4.1. Interpretation of Machine Learning Performance in MMSE Classification

The present study investigated the application of various machine learning algorithms for classifying cognitive status based on MMSE scores in community-dwelling older women. Our findings demonstrate that ensemble-based tree models, particularly CatBoost, GB, and AdaBoost, consistently outperformed other algorithms across multiple performance metrics. CatBoost achieved the highest weighted F1-score (87.05 ± 2.85%), while AdaBoost and GB reported superior precision-recall area under the curve (pr_auc) values of 92.49 ± 5.83% and 91.88 ± 5.18%, respectively. These results align with previous research indicating that gradient boosting algorithms often excel in handling structured clinical data with complex, nonlinear relationships [8,13].

The superior performance of CatBoost is particularly noteworthy given its robust handling of categorical variables without requiring extensive preprocessing, which is advantageous in clinical datasets where categorical features are common. The relatively low standard deviations observed for CatBoost, GB, and AdaBoost across repeated holdout experiments (w_f1 std < 3%) indicate their stability and consistent generalization capabilities, which are crucial for clinical applications where model reliability is paramount. In contrast, the Random Forest model exhibited higher variability (w_f1 std of 6.68%), suggesting greater sensitivity to specific data partitions—a finding consistent with previous comparative studies of tree-based ensemble methods [27,28].

Our results, showing the effectiveness of boosting algorithms in cognitive status classification, support the growing body of literature demonstrating the utility of these methods in healthcare applications. Recent studies have similarly found gradient boosting approaches to outperform traditional statistical methods in predicting various health outcomes, including cognitive decline. The consistent superiority of CatBoost in our study may be partially attributed to its innovative ordered boosting technique and effective handling of categorical features, which appears particularly well-suited for the mixed data types present in geriatric clinical assessments [9,27].

### 4.2. Clinical Significance of Physical Activity and Sedentary Behavior

The SHAP analysis revealed that physical activity metrics, particularly moderate physical activity minutes and walking days, emerged as consistently influential features across multiple high-performing models. This provides machine learning-based evidence supporting the well-established association between physical activity and cognitive health in older adults. Notably, higher values of moderate PA minutes and walking days generally corresponded to positive SHAP values in several models, suggesting an association with lower likelihood of severe cognitive impairment.

These results are consistent with previous findings [7], reporting that moderate physical activity is linked to reduced odds of sarcopenia in community-dwelling older women. The present findings extend this relationship to cognitive function, suggesting a potential interconnected pathway between sarcopenia, physical activity, and cognitive health in elderly women. This triad of relationships aligns with the “sarcopenia–cognitive impairment” hypothesis, which proposes shared biological mechanisms such as inflammation, oxidative stress, and neurotrophic factor dysregulation [3,4].

Conversely, longer sitting time minutes consistently demonstrated negative SHAP values in models like GB and CatBoost, indicating a potential adverse association with cognitive status. This is particularly significant, as it provides quantitative evidence for the detrimental impact of sedentary behavior on cognition, independent of physical activity levels. These results support epidemiological studies showing that prolonged sedentary time is associated with greater risk of cognitive decline, even among individuals who meet physical activity guidelines [29,30].

The observed negative association between prolonged sitting time and cognitive outcomes may not only reflect physical inactivity but also underlying motivational or affective dimensions. Apathy, defined as a multidimensional construct encompassing diminished goal-directed behavior, cognition, and emotional engagement, has been increasingly recognized as a key neuropsychiatric correlate of neurodegenerative conditions [31,32]. Reduced spontaneous movement or extended sedentary behavior may therefore represent behavioral manifestations of motivational decline rather than purely lifestyle choices. This interpretation suggests that interventions targeting cognitive health in older adults should consider integrating psychologically oriented strategies—such as behavioral activation or motivational coaching—alongside physical activity promotion to optimize outcomes.

The consistency of these findings across multiple high-performing machine learning models strengthens the evidence for physical activity and sedentary behavior as modifiable correlates of cognitive impairment in older women. This has important clinical implications, suggesting that interventions designed to promote moderate physical activity and reduce sedentary time may be associated with more favorable cognitive outcomes [33].

### 4.3. Anthropometric Factors and Cognitive Health

In addition to physical activity metrics, anthropometric variables including age, height, BMI, and weight consistently emerged as influential features in the SHAP analysis. The prominence of age as a predictor aligns with the well-established relationship between advancing age and increased risk of cognitive impairment. However, the significant contribution of BMI and weight to model predictions suggests a more complex relationship between body composition and cognitive health than previously recognized [6].

These findings resonate with the sarcopenia literature, as sarcopenia is often characterized by both loss of muscle mass and increased fat infiltration, which can affect BMI measurements in nonlinear ways. The fact that these anthropometric variables maintained consistent importance across models suggests potential underlying physiological mechanisms linking body composition to cognitive function. This supports the growing recognition of the “obesity-cognition paradox” in older adults, where both underweight and obesity have been associated with increased risk of cognitive decline [5,34].

The integration of physical activity metrics with anthropometric variables in predictive models represents a significant advancement over previous studies that typically examined these factors in isolation. Our machine learning approach has effectively captured the complex interplay between these factors, providing a more holistic understanding of their combined influence on cognitive status. This multidimensional perspective is crucial for developing comprehensive prevention and intervention strategies for cognitive impairment in older women [35].

While BMI emerged as one of the influential predictors in the SHAP analyses, its relationship with cognitive health is unlikely to be linear or uniform across individuals, particularly in older adults. BMI is a crude anthropometric metric that does not differentiate between adipose tissue and lean mass, and thus may obscure clinically relevant phenomena such as sarcopenic obesity—a condition characterized by excess body fat alongside reduced muscle strength and mass. Prior research suggests that higher BMI may exhibit both protective and detrimental effects depending on body composition, inflammatory status, and cardiometabolic profile, which may explain the modest yet complex contribution of BMI to cognitive performance observed in our models. Rather than functioning as an isolated risk factor, BMI likely interacts with muscle mass, physical function, and metabolic resilience to influence neurocognitive outcomes. Future studies employing dual-energy X-ray absorptiometry (DEXA) or bioimpedance-based body composition assessments would provide more precise insights into the mechanistic role of fat-to-lean mass distribution in cognitive aging.

Beyond the conventional interpretation of adiposity as a negative health determinant, recent systematic reviews have highlighted a potential “obesity paradox” in aging populations, whereby higher body mass may confer protective effects under certain physiological conditions. For instance, individuals with greater metabolic reserves or preserved muscle mass may be more resilient to neurodegenerative processes despite elevated BMI. Conversely, excess adiposity coupled with high inflammatory burden or reduced physical function may exacerbate cognitive deterioration. These divergent trajectories underscore the need to move beyond BMI as a unidimensional proxy and instead adopt body composition-based phenotyping approaches. Integrating measures such as waist-to-hip ratio, appendicular lean mass, or inflammatory biomarkers could refine risk stratification models and provide a more coherent understanding of adiposity-related cognitive resilience versus vulnerability.

### 4.4. Methodological Considerations and Model Interpretability

Although the methodological framework of this study incorporated widely used machine learning algorithms and SHAP-based interpretability techniques, these tools were not applied in a generic or algorithm-agnostic manner. Instead, several design decisions were deliberately tailored to the constraints of the dataset, including the choice of lightweight models to accommodate the limited sample size, the use of stratified repeated holdout validation to preserve class balance, and the prioritization of interpretable features to ensure clinical relevance. Rather than aiming for maximal predictive accuracy through complex architectures, the analytical strategy intentionally emphasized transparency, generalizability, and clinical plausibility—critical factors when modeling cognitive outcomes in small-scale geriatric populations.

The application of SHAP values for model interpretation represents a significant strength of this study, addressing the critical need for transparency in clinical machine learning applications. Unlike traditional feature importance measures that only indicate the relative contribution of features without directionality, SHAP values provide both the magnitude and direction of each feature’s influence on individual predictions. This enhanced interpretability is particularly valuable in clinical contexts where understanding the specific impact of risk factors on outcomes is essential for developing targeted interventions [10,36].

Our findings demonstrate how SHAP analysis can bridge the gap between high-performing ‘black-box’ models and clinically actionable insights. For instance, the color gradients in our SHAP violin plots clearly illustrate how increases in moderate physical activity generally lead to protective effects against severe cognitive impairment, while prolonged sitting time exerts detrimental effects. This level of interpretability transforms complex ensemble models from opaque predictors into tools that can inform clinical decision-making and patient counseling [37].

The consistent patterns observed across multiple high-performing models (CatBoost, GB, and AdaBoost) further strengthen the reliability of our feature importance findings. When multiple algorithms with different underlying mechanisms identify the same features as influential, it provides robust evidence for the genuine relevance of those features to the outcome of interest. This convergence of evidence from different modeling approaches represents a sophisticated validation method that goes beyond traditional statistical significance testing [38].

### 4.5. Limitations and Future Research Directions

Despite the promising findings, several limitations should be acknowledged. The relatively small sample size (67 participants) constrains the generalizability of our results, though the repeated holdout approach helped maximize data utility. The cross-sectional design prevents causal inference; therefore, all findings should be interpreted as associations rather than causal effects. The categorization of MMSE scores into two groups (≤17 vs. >17), while widely used in the literature to distinguish severe from mild cognitive impairment, reduces the granularity of cognitive function assessment. Additionally, our models did not incorporate potential mediating or moderating variables that could explain underlying mechanisms. Finally, focusing exclusively on community-dwelling older women limits generalizability to men or institutionalized populations, particularly given known sex differences in sarcopenia prevalence and cognitive aging trajectories.

Based on these limitations, we propose three prioritized research directions. First and most urgently, prospective longitudinal studies with larger sample sizes (300–500 participants) are needed to establish temporal and potentially causal relationships between physical activity patterns, body composition, and cognitive trajectories. Such studies should include repeated measures over 3–5 years follow-up, recruit diverse populations across both sexes and ethnic groups, and examine dose–response relationships between physical activity parameters and cognitive outcomes. Second, future research should incorporate biological markers and advanced imaging to elucidate underlying mechanisms, including inflammatory markers (IL-6, TNF-α, CRP), neurotrophic factors (BDNF, IGF-1), neuroimaging (structural MRI, functional MRI, PET scans) to assess brain structure and cerebral blood flow, and genetic data (e.g., APOE genotype) to examine gene–environment interactions. Third, to capture the full spectrum of cognitive function, future machine learning studies should treat MMSE as a continuous outcome or implement multi-class classification approaches, incorporate domain-specific cognitive assessments beyond MMSE (memory, executive function, processing speed), and validate models across multiple independent cohorts. These three priorities address the fundamental limitations of cross-sectional design, lack of mechanistic understanding, and binary classification, respectively, while providing a clear roadmap for advancing this field toward evidence-based interventions for cognitive health in aging populations.

While the present study offers valuable insights into cognitive health determinants among older women, the exclusive focus on a single sex limits the generalizability of the findings to the broader aging population. Evidence suggests that cognitive aging trajectories may differ between men and women, particularly in domains related to executive functioning, emotion recognition, and neuropsychiatric symptoms [39,40]. Biological factors such as hormone regulation, inflammatory profiles, and body composition, as well as sociocultural differences in health behaviors, may contribute to sex-specific mechanisms of cognitive decline. Therefore, future research should aim to include both sexes in order to elucidate potential interaction effects between sex, lifestyle factors, and cognitive performance, thereby enabling the development of more tailored intervention strategies.

Although the machine learning models yielded satisfactory performance in distinguishing cognitive impairment levels based on MMSE classification, the relatively small sample size (n = 67) warrants careful interpretation of the findings. Working with limited data inherently increases the risk of model overfitting, as algorithms may inadvertently learn patterns specific to the current sample rather than generalizable relationships applicable to broader populations. Repeated holdout validation was employed to partially mitigate this risk; however, this approach cannot fully substitute for external validation across independent cohorts. Moreover, more robust resampling techniques—such as bootstrapped confidence intervals or permutation-based stability assessments—were not feasible given the dataset constraints. Future studies should therefore aim to recruit larger and more heterogeneous populations, ideally including longitudinal follow-up to assess temporal consistency in prediction accuracy. Incorporating external replication datasets or bootstrapped resampling pipelines would further strengthen the reliability and clinical applicability of machine learning-based risk stratification in cognitive health research.

### 4.6. Clinical and Public Health Implications

The findings of this study have significant implications for clinical practice and public health interventions targeting cognitive health in older women. The consistent identification of moderate physical activity and walking frequency as protective factors suggests that relatively simple, accessible interventions could have meaningful impacts on cognitive outcomes. Clinicians should consider incorporating assessments of physical activity patterns and sedentary behavior into routine cognitive evaluations of older patients, and provide tailored recommendations for increasing moderate activity and reducing sitting time.

From a public health perspective, these findings support the development of community-based programs that promote physical activity specifically designed for older women. Given the emphasis on moderate-intensity activity in our results, interventions need not focus exclusively on high-intensity exercise, which may be inaccessible or unsafe for many older adults. Instead, programs emphasizing regular walking and other moderate activities could provide cognitive benefits while being more widely adoptable.

The integration of machine learning approaches into geriatric assessment represents a promising direction for personalized medicine. By identifying individual risk profiles based on physical activity patterns, anthropometric measures, and other factors, clinicians could develop targeted prevention strategies for cognitive decline. Our study demonstrates that ensemble boosting methods, particularly when combined with SHAP-based interpretation, can provide both accurate predictions and clinically interpretable insights—addressing a critical challenge in the adoption of artificial intelligence in healthcare settings.

## 5. Conclusions

This study demonstrates the superior performance of ensemble boosting algorithms, particularly CatBoost, in classifying cognitive status based on MMSE scores in older women. More importantly, the SHAP-based interpretation of these models revealed consistent patterns linking moderate physical activity, walking frequency, and reduced sedentary time with better cognitive outcomes, while highlighting the importance of anthropometric factors. These findings provide machine learning-validated evidence supporting the integration of physical activity assessment and promotion into cognitive health strategies for older women.

The convergence of evidence from multiple high-performing machine learning models strengthens the credibility of our findings and suggests robust relationships between physical activity patterns, body composition, and cognitive status. While acknowledging the limitations of our cross-sectional design and modest sample size, these results contribute to the growing body of evidence supporting physical activity as a modifiable protective factor against cognitive decline in older adults.

Future research should build upon these findings through longitudinal studies with larger, more diverse samples, incorporation of biological markers to elucidate underlying mechanisms, and development of targeted interventions based on machine learning-derived risk profiles. As the global population ages and the burden of cognitive impairment grows, the integration of advanced analytics with clinical gerontology represents a promising frontier for preserving cognitive health in later life.

## Figures and Tables

**Table 1:** Overview of Optimized Hyperparameters for Each Model.

Model Name	Hyperparameter Name	Hyperparameter Type	Hyperparameter Space
MLP	hidden_layer_sizes	Categorical	50 to 2000 (step = 50)
	Alpha	Real	High = 10^−1^, Low = 10^−4^
	Learning_rate	Real	High = 10^−1^, Low = 10^−4^
CatBoost	Learning_rate	Real	High = 0.3, Low = 10^−3^
	Iterations	Integer	High = 1500, Low = 100
	Depth	Integer	High = 10, Low = 4
	l2_leaf_reg	Real	High = 10, Low = 10^−3^
	loss_function	Categorical	‘Logloss’, ‘CrossEntropy’
LightGBM	Max_depth	Integer	High = 10, Low = 3
	Learning_rate	Real	High = 0.3, Low = 10^−2^
	n_estimators	Integer	High = 500, Low = 50
	Subsample	Real	High = 1.0, Low = 0.5
	colsample_bytree	Real	High = 1.0, Low = 0.5
	min_child_samples	Integer	High = 100, Low = 20
	reg_alpha	Real	High = 10, Low = 10^−3^
	reg_lambda	Real	High = 10, Low = 10^−3^
XGBoost	Max_depth	Integer	High = 10, Low = 3
	learning_rate	Real	High = 0.3, Low = 0.01
	n_estimators	Integer	High = 1500, Low = 50
	Subsample	Real	High = 1, Low = 0.5
	colsample_bytree	Real	High = 1, Low = 0.5
	Gamma	Real	High = 10, Low = 0
	min_child_weight	Integer	High = 10, Low = 1
RF	n_estimators	Integer	High = 1500, Low = 10
	max_depth	Integer	High = 20, Low = 2
	min_samples_split	Real	High = 0.99, Low = 0.01
	max_features	Real	High = 0.5, Low = 0.001
GB	n_estimators	Integer	High = 1500, Low = 50
	learning_rate	Real	High = 0.3, Low = 0.001
	max_depth	Integer	High = 30, Low = 1
	min_samples_split	Integer	High = 20, Low = 2
	min_samples_leaf	Integer	High = 20, Low = 1
LR	C	Real	High = 100, Low = 10^−6^
	Solver	Categorical	‘lbfgs’, ‘liblinear’
AdaBoost	n_estimators	Integer	High = 10, Low = 200
	learning_rate	Real	High = 0.01, Low = 2

**Table 2:** Mean performance scores and standard deviations obtained over 100 repeated holdout experiments for machine learning models used to classify MMSE status.

Model Name	w_f1 (%)	std (%)	acc (%)	std (%)	pre (%)	std (%)	rec (%)	std (%)	pr_auc (%)	std (%)	auc_roc (%)	std (%)
MLP	80.04	3.49	80.71	3.27	83.23	3.91	80.71	3.27	76.22	9.89	76.46	6.52
CatBoost	87.05	2.85	87.14	2.86	88.3	3.05	87.14	2.86	89.6	8.48	90	5.65
LightGBM	79.74	2.68	80	2.86	80.96	3.83	80	2.86	81.37	9.77	81.04	7.52
XGBoost	79.69	2.7	80	2.86	80.87	3.86	80	2.86	85.93	10.79	81.88	9.4
RF	81.84	6.68	82.14	6.59	84.99	6.44	82.14	6.59	88.34	9.07	85.94	7.94
GB	86.35	2.14	86.42	2.14	88.58	2.3	86.42	2.14	91.88	5.18	88.96	6.18
LR	77.45	3.69	77.86	3.85	78.91	4.86	77.86	3.85	80.21	8.51	78.96	5.39
AdaBoost	86.43	2.15	86.42	2.14	88.32	2.51	86.42	2.14	92.49	5.83	89.58	6.86

## Data Availability

The raw data supporting the conclusions of this article will be made available by the authors on request.
